# Efficacy and Safety of Tumor Treating Fields (TTFields) in Elderly Patients with Newly Diagnosed Glioblastoma: Subgroup Analysis of the Phase 3 EF-14 Clinical Trial

**DOI:** 10.3389/fonc.2021.671972

**Published:** 2021-09-27

**Authors:** Zvi Ram, Chae-Yong Kim, Andreas F. Hottinger, Ahmed Idbaih, Garth Nicholas, Jay-Jiguang Zhu

**Affiliations:** ^1^Department of Neurosurgery, Tel Aviv Medical Center and Tel Aviv University School of Medicine, Tel Aviv, Israel; ^2^Department of Neurosurgery, Seoul National University College of Medicine, Seoul, South Korea; ^3^Department of Clinical Neuroscience, CHUV Lausanne University Hospital & University of Lausanne, Lausanne, Switzerland; ^4^Service de Neurologie 2-Mazarin, Sorbonne Université, Inserm, CNRS, UMR S 1127, Institut du Cerveau, ICM, AP-HP, Hôpitaux Universitaires La Pitié Salpêtrière—Charles Foix, Service de Neurologie 2-Mazarin, Paris, France; ^5^Department of Medicine, University of Ottawa, Ottawa, ON, Canada; ^6^Department of Neurosurgery, University of Texas Health Science Center at Houston, Houston, TX, United States

**Keywords:** elderly patients, newly diagnosed glioblastoma, TTFields, Tumor Treating Fields, phase 3 clinical trial, efficacy and safety, quality-of-life, temozolomide

## Abstract

**Background:**

Understudied elderly patients comprise a large segment of high-risk patients with glioblastoma (GBM) that are challenging to treat. Tumor Treating Fields (TTFields) is a locoregional, noninvasive, antimitotic therapy delivering low-intensity, intermediate-frequency alternating electric fields to the tumor. In the phase 3 EF-14 clinical trial, TTFields (200 kHz) improved median progression-free survival (PFS) and median overall survival (OS) in patients with newly diagnosed GBM (ndGBM) when added concomitantly to maintenance temozolomide (TMZ). This EF-14 subgroup analysis evaluated the safety and efficacy of TTFields in elderly patients.

**Methods:**

All 134 patients who are ≥65 years of age were included (TTFields/TMZ combination, n=89; TMZ monotherapy, n=45; 2:1 ratio of randomization). PFS and OS were analyzed using Kaplan–Meier methodology (α=0.05). Health-related quality-of-life (HRQoL) was assessed using the European Organisation for Research and Treatment of Cancer (EORTC) quality-of-life questionnaire QLQ-C30 supplemented with the brain tumor module (QLQ-BN20). Adverse events (AEs) were evaluated using Common Terminology Criteria for AEs (CTCAE) v4.0.

**Results:**

The PFS was 6.5 months in patients randomized to the treatment group with TTFields/TMZ combination *versus* 3.9 months in patients treated with TMZ monotherapy (HR, 0.47; 95% CI, 0.30–0.74; *P*=0.0236). The OS was 17.4 months in patients treated with TTFields/TMZ combination *versus* 13.7 months in patients treated with TMZ monotherapy (HR, 0.51; 95% CI, 0.33–0.77; *P*=0.0204). Annual survival rates with TTFields/TMZ *versus* TMZ monotherapy were 39% (95% CI, 29–50%) *versus* 27% (95% CI, 15–41%; *P*=0.072) at 2 years, 19% (95% CI, 11–29%) *versus* 11% (95% CI, 4–23%; *P*=0.135) at 3 years, and 15% (95% CI, 7–25%) *versus* 0% at 5 years, respectively. There were no significant differences between groups in the preselected items of HRQoL assessment. Grade ≥3 systemic AEs were 46% in the TTFields/TMZ group *versus* 40% in the TMZ monotherapy group, without statistically significant difference between the two groups. The only TTFields-related AEs were reversible scalp skin reactions, with grades 1–2 and grade 3 skin reactions reported by 51% and 2% of patients, respectively.

**Conclusions:**

Combining TTFields with maintenance TMZ significantly improved PFS and OS in elderly patients with ndGBM in the phase 3 EF-14 clinical trial, without significant increases in systemic toxicity or negatively affecting patient HRQoL. TTFields-related skin AEs were low-grade and manageable.

**Clinical Trial Registration:**

https://clinicaltrials.gov/ct2/show/NCT00916409, identifier: NCT00916409.

## Introduction

Glioblastoma (GBM) is the most common type of primary malignant central nervous system tumor in adults (1, 2). GBM is an aggressive, incurable cancer, with 5-year survival rates of less than 10% (1, 3). In the United States (US), the overall incidence of GBM is 3.23 cases per 100,000, with a median age of 65 years at diagnosis (1). In patients who were 65–74 and 75–84 years of age, GBM incidence was 13.0 and 15.1 cases per 100,000, respectively, representing an approximately four times higher incidence in elderly patients compared to the overall population (1). As global populations age, the proportion of elderly patients with GBM relative to younger patients is predicted to increase (4, 5).

Management of elderly patients with GBM is challenging due to poor prognosis, frailty, comorbid conditions, and increased risk of adverse events (AEs) (4). The current treatment guidelines and standard of care (SOC) from the Society for Neuro-Oncology (SNO) and the European Association of Neuro-Oncology (EANO) for newly diagnosed GBM (ndGBM) is maximal safe tumor resection, followed by radiation therapy with concomitant and then adjuvant temozolomide (TMZ) chemotherapy, with emphasis on patients with *methylated O^6^-methylguanine-DNA methyltransferase (MGMT)* and good Karnofsky Performance Status (KPS). The same SOC remains the best option for elderly patients who are 65–70 years of age with good performance status (3, 6, 7). In patients who are ≥70 years of age that are candidates for radiochemotherapy, hypofractionated radiation therapy may be used (7, 8). In elderly patients with poor performance status, single modality treatment may be preferred: either SOC or hypofractionated radiation therapy, or TMZ for patients with *MGMT* promoter methylation status (4, 5, 7, 9). Tumor Treating Fields (TTFields; 200 kHz) is recommended for elderly patients as an adjunctive therapy option to maintenance TMZ (7).

TTFields is a locoregional treatment that inhibits mitosis and promotes apoptosis of proliferating cells through low-intensity, intermediate frequency alternating electric fields delivered *via* 2 pairs of arrays applied orthogonally to the skin of the scalp (10, 11). In preclinical studies, adding TTFields to chemotherapy demonstrated an additive antiproliferative effect in human GBM cell lines and in animal tumor models (12). In the randomized phase 3 EF-14 trial, TTFields (200 kHz) in combination with maintenance TMZ chemotherapy significantly improved survival outcomes in patients with ndGBM compared to TMZ alone (13), without leading to a decline in quality-of-life, except for more itchy skin (14, 15). Additionally, subgroup analysis indicated that survival benefits were greater and positively correlated with higher TTFields usage rates (16).

This subgroup analysis from the EF-14 trial reports on the efficacy and safety of TTFields (200 kHz) in combination with maintenance TMZ in elderly patients with ndGBM.

## Materials and Methods

EF-14 (NCT00916409) was a multicenter, phase 3, open-label, randomized clinical trial that assessed the efficacy and safety of TTFields (200 kHz) plus maintenance TMZ combination *versus* maintenance TMZ monotherapy in 695 patients with ndGBM (13). All patients provided written informed consent prior to entering the study. Patients with newly diagnosed, histologically confirmed supratentorial GBM, with a KPS score of ≥70, and who were progression-free following debulking surgery or biopsy and standard radiotherapy with concomitant TMZ were eligible to enroll. Subjects were randomized 2:1 to receive TTFields plus TMZ chemotherapy or TMZ monotherapy. TMZ was administered to both groups (150–200 mg/m^2^) for 5 days per 28-day cycle (6–12 cycles) (13). TTFields (200 kHz) was delivered from a portable device (Optune^®^, Novocure^®^ GmbH, device manufacturer) *via* 4 panel arrays with 9 ceramic disks of each panel placed on the shaved scalp for a recommended monthly average usage of ≥18 h/day. TTFields therapy was initiated between 4 and 7 weeks after completion of radiotherapy (13). Full eligibility criteria and details of the EF-14 trial protocol have been previously published (13).

**Figure 1:**
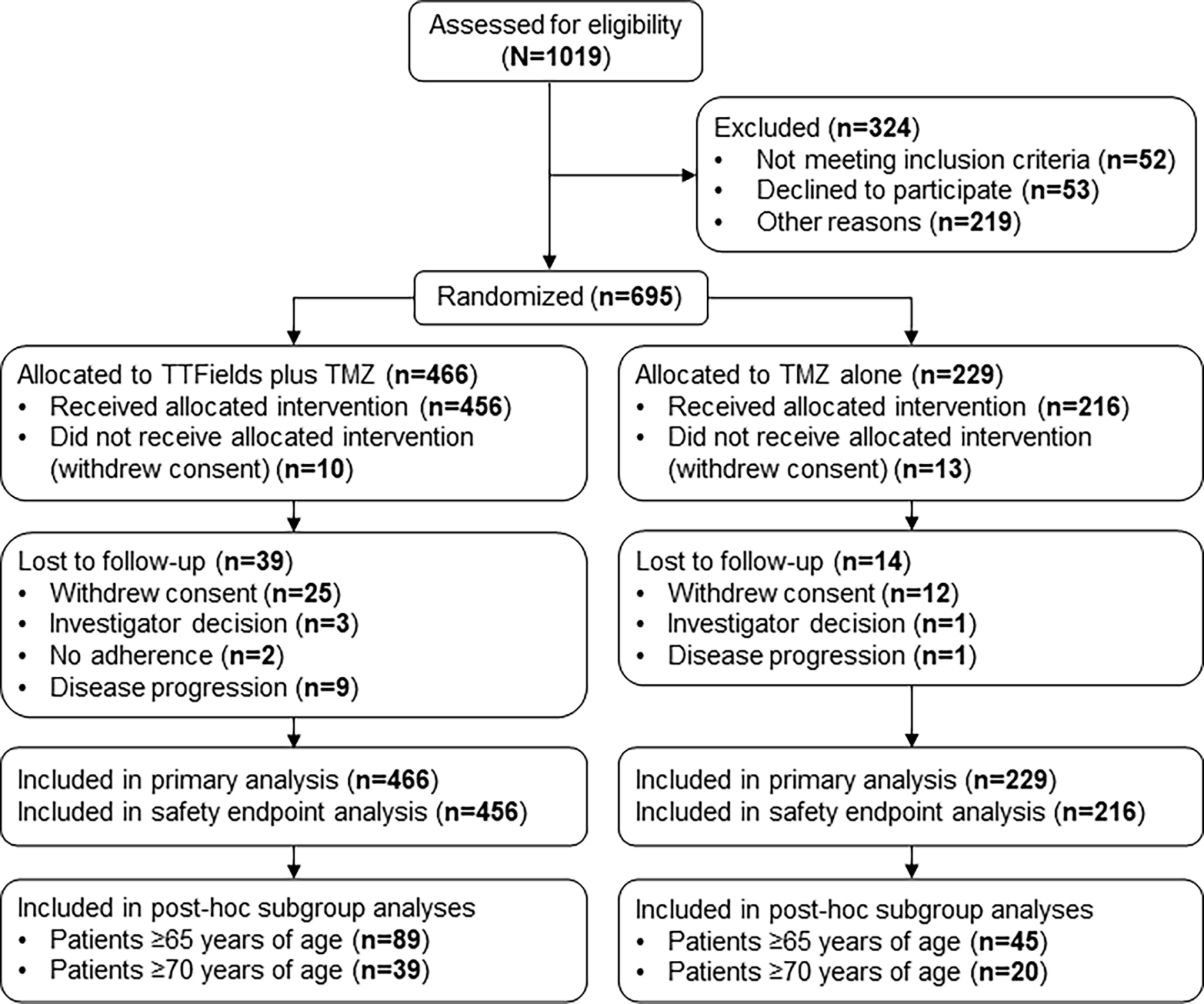
CONSORT Diagram. Updated from Stupp R, Taillibert S, Kanner A, Read W, Steinberg DM, Lhermitte B, et al. Effect of tumor-treating fields plus maintenance temozolomide *vs* maintenance temozolomide alone on survival in patients with glioblastoma a randomized clinical trial. *JAMA*. (2017) 318:2306–16. TMZ, temozolomide; TTFields, Tumor Treating Fields.

This subgroup analysis included 134 patients who were ≥65 years of age from the phase 3 EF-14 trial (TTFields plus TMZ chemotherapy, n=89; TMZ monotherapy, n=45) (**Figure 1**). Following the interim analysis of the intent-to-treat population, a total of 7 patients from the TMZ monotherapy group who were ≥65 years of age were crossed over to the TTFields plus TMZ combination group. However, these patients were analyzed in the TMZ monotherapy group as originally randomized. The primary efficacy endpoint in the EF-14 trial was progression-free survival (PFS), defined as the time from the start of the study treatment (randomization) to disease progression or death. PFS was assessed by an independent central radiological review. The secondary endpoint in the EF-14 trial was overall survival (OS), measured from study treatment start (randomization) until death or the time at which the patient was censored (on the last date they were known to be alive). Other efficacy endpoints included PFS at 6 months (PFS-6), annual survival rates, and health-related quality-of-life (HRQoL). The OS and PFS were also evaluated in the small cohort of advanced age elderly patients who are ≥70 years of age (n=59) to suffice global definitions of elderly and since GBM incidence has been observed to be increasing in this age group compared to other age groups over the last few decades due to progressive aging (17). Active assessment of treatments regarding efficacy, safety, and QoL outcomes in elderly is a great unmet need due to older age being a negative prognostic factor. However, considering the projected increase in this elderly group, age alone should no longer be a reason to give palliative care rather than treatment as is the current tendency (17).

The threshold for TTFields duration of usage was defined as the monthly average of TTFields daily usage of ≥75% (≥18 h/day), which was calculated as the average number of hours per day that TTFields therapy was used during all treatment cycles (i.e., between cycle 1 and cycle 6). PFS and OS were analyzed in patients who were stratified by TTFields daily usage of <75% *vs* ≥75% (i.e., <18 h/day *vs* ≥18 h/day).

AEs were recorded prospectively according to the National Cancer Institute’s Common Terminology Criteria for AEs (NCI-CTCAE version 4.0) and are presented descriptively as the number and percentage of cases for each AE term. HRQoL was assessed using the European Organisation for Research and Treatment of Cancer (EORTC) quality-of-life questionnaire QLQ-C30 supplemented with the brain tumor module (QLQ-BN20). Patients completed questionnaires every 3 months, with analysis performed on questionnaires completed during the first 12 months of the trial. The 9 preselected, questionnaire items that were deemed most relevant for TTFields-treated patients were as follows: 1) global health status, 2) physical functioning, 3) cognitive functioning, 4) role functioning, 5) social functioning, 6) emotional functioning, 7) itchy skin, 8) pain, and 9) weakness of legs. Outcomes were deterioration-free survival (DFS) and time-to-deterioration (TTD) as previously described (14). DFS was defined as the time to achieve at least a 10-point reduction in scores from baseline without a subsequent improvement of ≥10 points, progressive disease, or death. TTD was defined using the same aforementioned criteria but excluded progressive disease.

### Statistical Analysis

Kaplan–Meier survival analysis with stratified log-rank testing to compare treatment groups (α=0.05) was used to analyze PFS and OS. Patients lost to radiological follow-up were censored at the last date they were known to be alive and progression-free (i.e., PFS) or alive (i.e., OS). Kaplan–Meier estimates of the survival rates at 6 months and annually were compared between groups using a one-sided Z distribution test. Cox proportional hazards modeling was used to analyze PFS and OS, controlling for the treatment group, age, *MGMT* methylation status, extent of resection, KPS, and country (US *versus* all other countries). The threshold for significant interactions in the model was specified at α=0.05. Kaplan–Meier methodology was used to estimate DFS and TTD distributions and median times. The number and percentage of cases for each AE are presented for patients who received ≥1 dose of maintenance TMZ or 1 day of TTFields therapy. All analyses were performed using Statistical Analysis System (SAS) version 9.4 (Cary, NC).

## Results

### Baseline Characteristics of Elderly Patients

Patient baseline characteristics and known prognostic factors for elderly patients ≥65 years of age were balanced between treatment groups (**Table 1**). Patients were 69 years of age (range, 65–83) and 68 years of age (range, 65-80) for the TTFields plus TMZ combination group and the TMZ monotherapy group, respectively. In the TTFields plus TMZ combination group, 70% of the patients were male, and in the TMZ monotherapy group, 69% of the patients were male, which are both representative of the real-world GBM population (18, 19). The median KPS score was 90; although this is high, it was balanced between treatment groups. Across all patients, 54% had undergone a gross total resection and 13% had a diagnostic biopsy only. Tumor tissue samples were available from 83% of the patients for *MGMT* testing. Of valid tests, 41% of the patients were *MGMT* promoter methylation status positive (40%, TTFields/TMZ combination group; 44%, TMZ monotherapy group). Of the patients with available samples, 3% demonstrated *isocitrate dehydrogenase-R132H (IDH1^R132H^)* mutant tumors (4%, TTFields/TMZ combination group; 0%, TMZ monotherapy group). The baseline characteristics for elderly patients who are ≥70 years of age are shown in **Table 2**.

**Table 1:** Patient baseline characteristics in TTFields (200 kHz) plus TMZ combination *versus* TMZ monotherapy groups for patients ≥65 years of age.

Characteristics	TTFields plus TMZ (n = 89)	TMZ alone (n = 45)	*P* value*
Age, years, median (range)	69 (65–83)	68 (65–80)	0.339
Sex, n (%)			0.927
Male	62 (70)	31 (69)	
Female	27 (30)	14 (31)	
Corticosteroid therapy, n (%)	28 (32)	10 (22)	0.262
Extent of resection, n (%)			0.499
Biopsy	13 (15)	4 (9)	
Partial resection	31 (35)	14 (31)	
Gross total resection	45 (51)	27 (60)	
*MGMT* tissue available and tested, n (%)	75 (84)	36 (80)	0.761
Methylated	30 (40)	16 (44)	
Unmethylated	38 (51)	18 (50)	
Invalid	7 (9)	2 (6)	
*IDH1^R132H^* tissue available and tested, n (%)	53 (60)	23 (51)	0.345
Positive	2 (4)	0 (0)	
Negative	51 (96)	23 (100)	
EGFR tissue available and tested, n (%)	53 (60)	21 (47)	0.396
Amplified	22 (42)	11 (52)	
Not amplified	31 (58)	10 (48)	
Chromosomes 1p and 19q tissue available and tested, n (%)	51 (57)	23 (51)	0.419
Codeletion	1 (2)	0 (0)	
Loss 1p only	1 (2)	1 (4)	
Loss 19q only	0 (0)	1 (4)	
Retained	47 (92)	21 (91)	
Invalid	2 (4)	0 (0)	
KPS,^a^ median (range)	90 (60–100)	90 (70–100)	
Time from diagnosis to randomization, months, median (range)	3.8 (2.6–5.7)	3.8 (1.4–5.4)	0.770
Time from last day of radiotherapy to randomization, days, median (range)	37 (23–54)	40 (29–51)	0.065
TMZ cycles until first tumor progression, n, median (range)	6.0 (1–18)	5.5 (1–20)	*NA*
Time from randomization to TTFields initiation, days, median (range)	5.5 (1–14)	*NA*	*NA*
Duration of TTFields therapy, months, median (range)	7.9 (0–20)	*NA*	*NA*
TTFields daily usage ≥75%, n (%)	51 (57)	*NA*	*NA*

EGFR, epidermal growth factor receptor gene; IDH1^R132H^, isocitrate dehydrogenase gene 1 R132H mutation site; KPS, Karnofsky Performance Score; MGMT, O^6^-methylguanine-DNA-methyltransferase gene; n, number of patients; NA, not applicable; TMZ, temozolomide; TTFields, Tumor Treating Fields.

a Karnofsky Performance Score is measured from 0 to 100 in 10-point bins. A higher score represents better performance status.

*Chi-squared test for percentage values and T test for mean values.

Total percentage sums may not equal 100 or total percent of a patient subpopulation due to rounding to the nearest integer.

**Table 2:** Baseline characteristics in TTFields (200 kHz) plus TMZ combination versus TMZ monotherapy groups for patients ≥70 years of age.

Characteristics	TTFields plus TMZ (n=39)	TMZ alone (n=20)	*P* value*
Age, years, median (range)	74 (70–83)	73 (70–80)	0.186
Sex, n (%)			0.957
Male	29 (74)	15 (75)	
Female	10 (26)	5 (25)	
Corticosteroid therapy, n (%)	12 (31)	5 (25)	0.643
Extent of resection, n (%)			0.312
Biopsy	7 (18)	1 (5)	
Partial resection	13 (33)	6 (30)	
Gross total resection	19 (49)	13 (65)	
*MGMT* tissue available and tested, n (%)			0.443
Methylated	16 (46)	4 (27)	
Unmethylated	15 (43)	9 (60)	
Invalid	4 (11)	2 (13)	
*IDH1^R132H^* tissue available and tested, n (%)	24 (62)	10 (50)	0.512
Positive	1 (4)	0 (0)	
Negative	23 (96)	10 (100)	
EGFR tissue available and tested, n (%)	26 (67)	10 (50)	0.529
Amplified	10 (38)	5 (50)	
Not amplified	16 (62)	5 (50)	
Chromosomes 1p and 19q tissue available and tested, n (%)	24 (62)	10 (50)	0.241
Codeletion	0 (0)	0 (0)	
Loss 1p only	0 (0)	0 (0)	
Loss 19q only	0 (0)	1 (10)	
Retained	23 (96)	9 (90)	
Invalid	1 (4)	0 (0)	
KPS,^a^ median (range)	85 (60–100)	90 (70–100)	
Time from diagnosis to randomization, months, median (range)	3.7 (2.6–5.1)	3.9 (2.8–5.4)	0.268
Time from last day of radiotherapy to randomization, days, median (range)	35 (23–49)	42 (29–50)	0.016
TMZ cycles until first tumor progression, n, median (range)	6 (1–15)	6 (1–12)	0.441
Time from randomization to TTFields initiation, days, median (range)	5 (1–13)	*NA*	
Duration of TTFields therapy, months, median (range)	6.9 (0–40)	*NA*	*NA*
TTFields daily usage ≥75%, n (%)	19 (41)	*NA*	*NA*

EGFR, epidermal growth factor receptor gene; IDH1^R132H^, isocitrate dehydrogenase gene 1 R132H mutation site; KPS, Karnofsky Performance Score; MGMT, O^6^-methylguanine-DNA-methyltransferase gene; n, number of patients; NA, not applicable; TMZ, temozolomide; TTFields, Tumor Treating Fields.

a Karnofsky Performance Score is measured from 0 to 100 in 10-point bins. A higher score represents better performance status.

*Chi-squared test for percentage values and T test for means values.

Total percentage sums may not equal 100 or total percent of a patient subpopulation due to rounding to nearest integer.

The median time from histological diagnosis to randomization was 3.8 months (including 6 weeks concurrent radiation therapy with TMZ) in both groups. The median time from end of radiation therapy to randomization was 37 days in the TTFields plus TMZ combination group and 40 days in the TMZ monotherapy group. The median time from randomization to TTFields initiation was 5.5 days (range, 1–14 days). The median number of TMZ cycles until first tumor progression was 6 cycles (range, 1–18 cycles) and 5.5 cycles (range, 1–20 cycles) for patients in the TTFields plus TMZ combination group and the TMZ monotherapy group, respectively. The median duration of TTFields treatment was 7.9 months (range, 0–20 months).

### Efficacy

**Figure 2:**
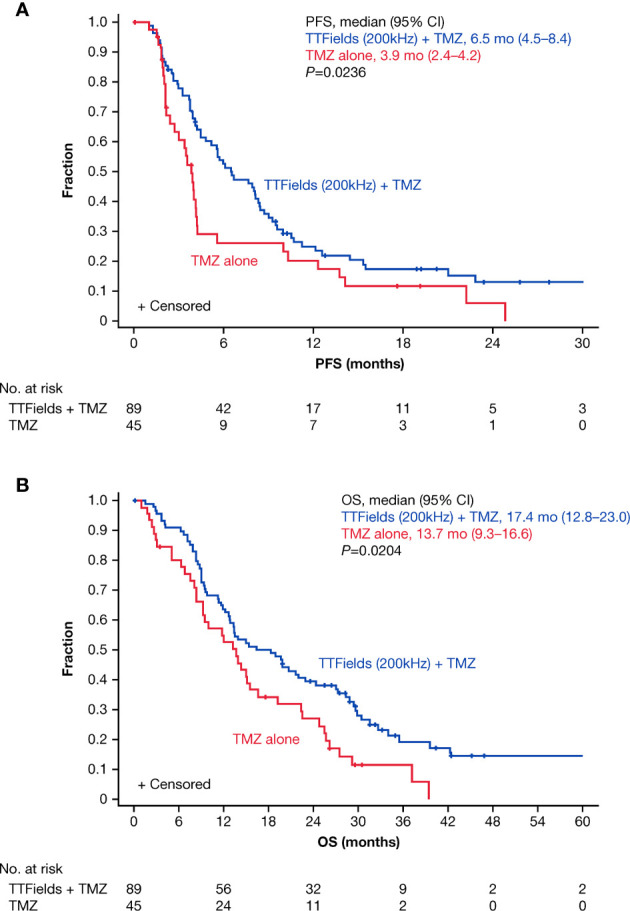
Kaplan–Meier curves of **(A)** PFS and **(B)** OS of elderly patients ≥65 years of age who were treated with TTFields plus TMZ combination *(blue line)* compared to TMZ monotherapy (red line). CI, confidence interval; mo, month; no, number; OS, overall survival; PFS, progression-free survival; TMZ, temozolomide; TTFields, Tumor Treating Fields.

Median duration to follow-up was 8 months for the TTFields plus TMZ combination *versus* 7 months for the TMZ monotherapy group. At the time of assessment, 20 patients were alive and 69 patients had died (not including 20 censored patients) for the TTFields plus TMZ group. There were no patients who were alive (not including 5 censored patients) at the time of assessment for the TMZ group. The PFS was 6.5 months (95% confidence interval [CI], 4.5–8.4 months) with TTFields plus TMZ combination *versus* 3.9 months (95% CI, 2.4–4.2 months) with TMZ monotherapy in patients ≥65 years of age (HR, 0.47; 95% CI, 0.30–0.74; *P*=0.0236) (**Figure 2A** and **Table 3**). In addition, OS was 17.4 months (95% CI, 12.8–23.0 months) in the TTFields plus TMZ combination group *versus* 13.7 months (95% CI, 9.3–16.6 months) in the TMZ monotherapy group (HR, 0.51; 95% CI, 0.33–0.77; *P*=0.0204) (**Figure 2B** and **Table 3**).

**Table 3:** Summary of efficacy outcomes comparing TTFields plus TMZ combinations *versus* TMZ monotherapy in patients ≥65 years of age.

	TTFields plus TMZ (n = 89)	TMZ alone (n = 45)	*P* value; HR (95% CI)
**PFS** (95% CI), months	6.5 (4.5–8.4)	3.9 (2.4–4.2)	*P*=0.0236^a^ 0.47 (0.30–0.74)
**OS** (95% CI), months	17.4 (12.8–23.0)	13.7 (9.3–16.6)	*P*=0.0204^a^ 0.51 (0.33–0.77)
**PFS-6**, % (95% CI)	52.5 (41.0–62.8)	26.1 (13.1–41.1)	*P*=0.002^a^
**Annual survival rates** ^b^, % (95% CI)			
1 year	63.6 (52.7–72.7)	52.5 (36.9–65.9)	*P*=0.110^c^
2 years	39.4 (29.2–49.5)	26.9 (14.8–40.6)	*P*=0.072^c^
3 years	19.1 (10.8–29.3)	11.4 (3.9–23.4)	*P*=0.135^c^
4 years	14.6 (7.0–24.8)	0	*NA*
5 years	14.6 (7.0–24.8)	0	*NA*

CI, confidence interval; HR, hazard ratio; NA, not applicable; OS, overall survival; PFS, progression-free survival; PFS-6, progression-free survival rate at 6 months; TMZ, temozolomide; TTFields, Tumor Treating Fields.

Survival rates are actuarial estimates according to the Kaplan–Meier method. Hazard ratios were calculated for only the primary and secondary endpoints.

a Log-rank test.

b Annual survival rates are given at 1–5 years after randomization.

c One-sided Z distribution test.

Total percentage sums may not equal 100 or total percent of a patient subpopulation due to rounding to the nearest integer.

PFS-6 in TTFields/TMZ-treated elderly patients ≥65 years of age was 52.5% (95% CI, 41.0–62.8%) *versus* 26.1% (95% CI, 13.1–41.1%) in TMZ alone-treated patients (*P*=0.002) (**Table 3**). Annual survival rates in patients treated with TTFields plus TMZ *versus* TMZ alone were 39.4% (95% CI, 29.2–49.5%) *versus* 26.9% (95% CI, 14.8–40.6%) (*P*=0.072) at 2 years, 19.1% (95% CI, 10.8–29.3%) *versus* 11.4% (95% CI, 3.9–23.4%) (*P*=0.135) at 3 years, and 14.6% (95% CI, 7.0–24.8%) *versus* 0% at 5 years, respectively (**Table 3**).

In patients ≥70 years of age (n=59), OS was 14.0 months in the TTFields plus TMZ combination group *versus* 8.2 months in the TMZ monotherapy group (HR, 0.29; 95% CI, 0.15–0.60; *P*=0.0175). Furthermore, PFS was 6.7 months *versus* 3.5 months with TTFields plus TMZ *versus* TMZ alone (HR, 0.28; 95% CI, 0.13–0.59; *P*=0.0011) in patients who were ≥70 years of age.

Analyses of PFS and OS using Cox proportional hazard modeling with KPS, *MGMT* promoter methylation status, geographic region, age, tumor location, and resection status as covariates supported the primary findings in patients who were ≥65 years of age. TTFields plus TMZ combination treatment (HR, 0.44; 95% CI, 0.28–0.68; *P*<0.001), presence of *MGMT* promoter methylation status (HR, 0.46; 95% CI, 0.28–0.75; *P*=0.0002), younger age (as a continuous variable; HR, 1.07 per year; 95% CI, 1.02–1.10; *P*=0.010), and higher KPS (as a categorical variable in 10 point increments; *P*=0.013) were associated with longer OS. Sex, tumor location, geographic region, and extent of resection were not associated with a significant difference in OS (all *P*>0.05). Similar results for PFS were observed, except that age was not a significant prognostic factor for PFS.

**Figure 3:**
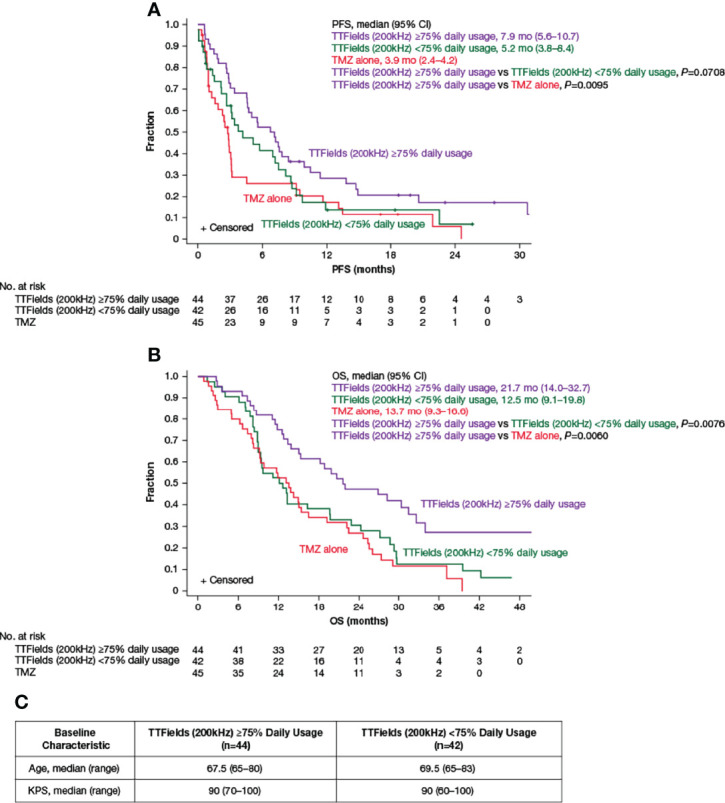
Kaplan–Meier curves of **(A)** median PFS and **(B)** median OS of TTFields plus TMZ combination with TTFields daily usage of ≥75% *(purple lines)* compared to TTFields daily usage of <75% *(green lines)* and TMZ alone *(red lines).* **(C)** Baseline age and KPS of patients ≥65 years of age with TTFields daily usage of ≥75% and <75%. CI, confidence interval; KPS, Karnofsky Performance Status; mo, month; no, number; OS, overall survival; PFS, progression-free survival; TMZ, temozolomide; TTFields, Tumor Treating Fields.

Approximately 57% of elderly patients ≥65 years of age achieved TTFields daily usage of ≥75% (≥18 h/day). The PFS was 7.9 months (95% CI, 5.6–10.7 months) in patients with ≥75% daily usage *versus* 5.2 months (95% CI, 3.8–8.4 months) in patients with <75% daily usage (HR, 0.69; 95% CI, 0.42–1.10; *P*=0.0708) (**Figure 3A** and **Table 4**). Patients with ≥75% daily usage had a significantly longer OS (21.7 months; 95% CI, 14.0–32.7 months) relative to patients with <75% daily usage (12.5 months; 95% CI, 9.1–19.8 months; HR, 0.52; 95% CI, 0.32–0.86; *P*=0.0076) (**Figure 3B** and **Table 4**). Overall, median (ranges) for age and KPS were comparable across both the ≥75% (≥18 h/day) and the <75% (≥18 h/day) daily usage subgroups (**Figure 3C**).

**Table 4:** Summary of **(A)** PFS and OS endpoints and **(B)** other efficacy endpoints by TTFields daily usage in patients ≥65 years of age.

	TTFields plus TMZ		TTFields daily usage of ≥75% *(TTFields plus TMZ vs TMZ alone)*	
	TTFields daily usage^a^ ≥75% (n = 44)	TTFields daily usage^a^ <75% (n = 42)	HR (95% CI) *P* value	HR (95% CI) *P* value
**A**				
**PFS**, median (95% CI), months	7.9 (5.6–10.7)	5.2 (3.8–8.4)	0.69 (0.42–1.10) 0.0708^b^	0.53 (0.33–0.86) 0.0095^b^
**OS**, median (95% CI), months	21.7 (14.0–32.7)	12.5 (9.1–19.8)	0.52 (0.32–0.86) 0.0076^b^	0.44 (0.27–0.72) 0.006^b^
**B**				
		**TTFields plus TMZ**		***P* value**
	**TTFields daily usage** ^a^ **≥75% (n=44)**		**TTFields daily usage** ^a^ **<75% (n=42)**	
**PFS-6**				
PFS-6, % (95% CI)	56.8 (41.0–69.9)		47.4 (30.4–62.5)	0.200^d^
**Annual survival rates**^**c**^				
1 year, **%** (95% CI)	75.0 (59.4–85.3)		52.4 (36.4–66.1)	0.013^d^
2 years, **%** (95% CI)	47.3 (32.1–61.2)		30.5 (17.3–44.7)	0.053^d^
3 years, **%** (95% CI)	27.1 (13.3–43.0)		12.3 (4.1–25.3)	0.062^d^
4 years, **%** (95% CI)	27.1 (13.3–43.0)		0	*NA*
5 years, **%** (95% CI)	27.1 (13.3–43.0)		0	*NA*

CI, confidence interval; HR, hazard ratio; NA, not applicable; OS, overall survival; PFS, progression-free survival; PFS-6, progression-free survival rate at 6 months; TMZ, temozolomide; TTFields, Tumor Treating Fields.

a Mean TTFields (200 kHz) usage was calculated as the average value for all treatment cycles (between cycle 1 and cycle 6). The usage threshold specified in this analysis was TTFields daily usage of ≥75% (i.e., ≥18 h/day).

b Log-rank test.

c Annual survival rates are given at 1–5 years after randomization.

d One-sided Z distribution test.

Survival rates are actuarial estimates according to the Kaplan–Meier method. Hazard ratios were calculated for only the primary and secondary endpoints.

Total percentage sums may not equal 100 or total percent of a patient subpopulation due to rounding to the nearest integer.

### Adverse Events and Tolerability

The addition of TTFields therapy to maintenance TMZ chemotherapy in elderly patients ≥65 years of age was not associated with any differences in the incidence of systemic AEs (AEs grade ≥3: 46% *versus* 40%, respectively; *P*=0.3518) (**Table 5**). Serious AEs (SAEs) were reported by 39% and 33% of patients in the TTFields plus TMZ combination group and the TMZ monotherapy group, respectively. No SAEs were reported as related to treatment with TTFields therapy. The overall incidence and severity of AEs observed were comparable between the 2 treatment groups. Localized skin reactions (medical device site skin reaction beneath the arrays) were reported by 53% of patients treated with TTFields plus TMZ combination. Skin reactions beneath the arrays were mild-to-moderate (grade 1–2) in 51% of the patients and severe (grade 3) in 2% of the patients. No other AEs were reported that were significantly more frequent in elderly patients treated with TTFields plus TMZ relative to TMZ alone.

**Table 5:** Grades 3–4 adverse events (AEs) with ≥5% incidence in any treatment group in patients ≥65 years of age.

NCI-CTCAE Version 4.0, by system organ class/preferred term	Grade 3–4 AEs, n (%)	
	TTFields plus TMZ (n = 87)	TMZ alone (n = 42)
**Number of patients with ≥ 1 AE**	40 (46)	17 (40)
**Blood and lymphatic system disorders**	10 (11)	5 (12)
Lymphopenia	4 (5)	1 (2)
Thrombocytopenia	6 (7)	4 (10)
**General disorders and administration site conditions**	12 (14)	2 (5)
Asthenia	6 (7)	1 (2)
Fatigue	5 (6)	1 (2)
**Injury, poisoning, and procedural complications (falls and medical device site reactions)**	5 (6)	0 (0)
Investigations	3 (3)	2 (5)
Platelet count decreased	3 (3)	2 (5)
**Metabolism and nutrition disorders**	1 (1)	2 (5)
Hyperglycemia	1 (1)	2 (5)
**Musculoskeletal and connective tissue disorders**	5 (6)	0 (0)
**Nervous system disorders**	14 (16)	7 (17)
Cognitive disorder	2 (2)	2 (5)
Convulsion	5 (6)	3 (7)
Hemiparesis	4 (5)	1 (2)
**Respiratory, thoracic, and mediastinal disorders**	4 (5)	3 (7)
Pulmonary embolism	3 (3)	3 (7)

AE, adverse event; NCI-CTCAE, National Cancer Institute—Common Terminology Criteria for Adverse Events; TMZ, temozolomide; TTFields, Tumor Treating Fields.

The safety population includes all patients who received ≥1 dose of maintenance TMZ or ≥1 day of treatment with TTFields (200 kHz).

**Figure 4:**
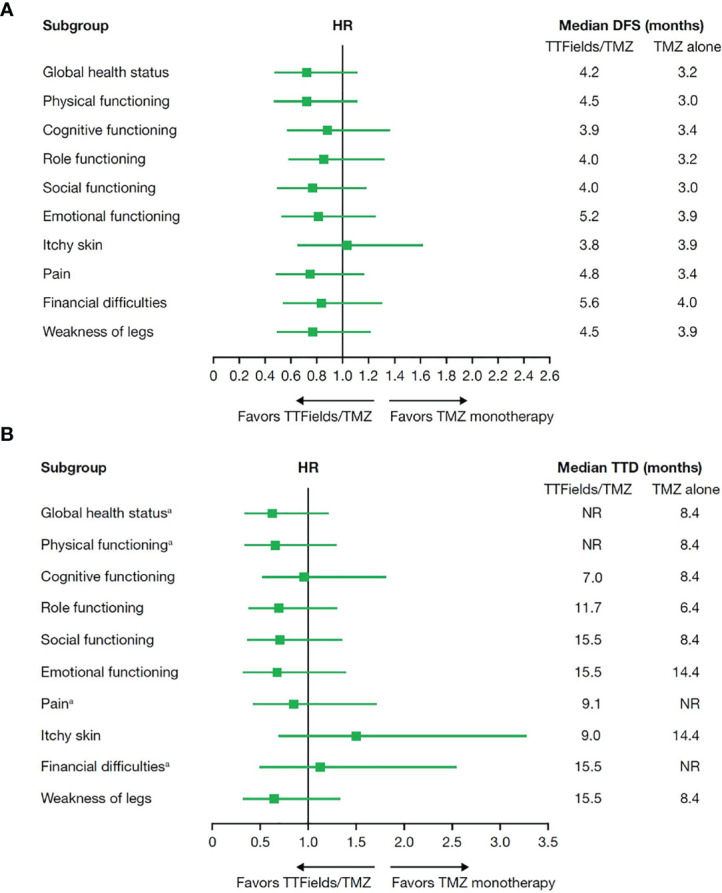
**(A)** Median deterioration-free survival and **(B)** median time to deterioration for health-related quality-of-life domains in patients ≥65 years of age who received TTFields plus TMZ combination compared with TMZ monotherapy. DFS, deterioration-free survival; HR, hazard ratio; NR, not reached; TMZ, temozolomide; TTD, time-to-deterioration; TTFields, Tumor Treating Fields. ^a^At the time of this analysis, median TTD was not reached for global health status and physical functioning in the TTFields plus TMZ combination group or pain and financial difficulties in the TMZ monotherapy group.

To estimate patient tolerability, the impact of treatment with TTFields on activities of daily life and cognition was analyzed using quality-of-life EORTC QLQ-C30 and QLQ-BN20 questionnaires. As expected, patient adherence to HRQoL assessments decreased from 91.0% at baseline to 39.2% at 12 months of follow-up for the TTFields plus TMZ combination group and dropped from 88.9% at baseline to 39.1% at 12 months of follow-up for the TMZ monotherapy group with a 2:1 ratio maintained between the combination and monotherapy groups (**Table 6**). No statistically significant differences in DFS or TTD were observed between treatment groups for any of the preselected items (**Figure 4**).

**Table 6:** Compliance of patients ≥65 years of age in performing QoL questionnaires.

	No. of patients completing questionnaire n/N^a^ (%)	
	TTFields plus TMZ	TMZ alone
**Baseline**	81/89 (91)	40/45 (89)
**Month 3**	55/85 (65)	20/39 (51)
**Month 6**	43/80 (54)	18/35 (51)
**Month 9**	23/68 (34)	14/29 (48)
**Month 12**	22/56 (39)	9/23 (39)

No., number; QoL, quality-of-life; TMZ, temozolomide; TTFields, Tumor Treating Fields.

a Total number of patients (N) excludes deaths and patients censored prior to designated time point.

## Discussion

In this subgroup analysis of elderly patients who are ≥65 years of age from the EF-14 clinical trial, the addition of TTFields to maintenance TMZ significantly improved PFS (6.5 *vs* 3.9 months) and OS (17.4 *vs* 13.7 months) compared to maintenance TMZ therapy alone. Overall, compared to other treatments that have been demonstrated to be toxic and with limited administration in this patient population, such as radiotherapy and TMZ, TTFields therapy is well tolerated and can be used continuously in patients with ndGBM, including elderly.

A significantly longer OS was observed in patients with an average daily usage of TTFields of ≥75% (≥18 h/day) relative to those with a daily usage of <75% (21.7 months *vs* 12.5 months). This finding is consistent with the overall EF-14 trial population and a study of TTFields in recurrent GBM (rGBM; phase 3 EF-11 trial), as well as in a post-approval registry study (PriDe) of patients with rGBM, thus potentially signifying the importance of increased usage of TTFields therapy to optimize survival outcomes in patients with GBM (16, 20, 21). However, it is still to be determined whether compliance to TTFields usage recommendations (≥18 h/day) drives patient response independently of other prognostic factors. In this analysis, more than half of elderly patients achieved an average daily usage of ≥75% (≥18 h/day), suggesting that elderly patients are able to comply with usage recommendations and that TTFields therapy is therefore feasible in this population. Moreover, improvements in PFS and OS with adjunctive TTFields were observed in the small subgroup of elderly patients who were ≥70 years of age, which is an age group most likely to have clinically relevant and potentially treatment-limiting age-related comorbidities and frailty (4, 5).

Since patients with tumor progression within 3 months of diagnosis were excluded from the EF-14 trial, this analysis likely included patients with a relatively better prognosis compared to trials that included patients from diagnosis. However, of note, both PFS and OS were calculated from randomization rather than from histological diagnosis. The median time from diagnosis to randomization was 3.8 months in both groups. The median time from radiotherapy to randomization was 37 and 40 days for TTFields/TMZ combination therapy and TMZ monotherapy, respectively, and the median time from randomization to TTFields therapy initiation was 5.5 days. Hence, an additional 3.8 months should be added to OS times, supporting the notion that elderly patients who are able to complete radiation therapy and are candidates for maintenance therapy may show extended PFS and OS benefit and should be offered all available treatment strategies, including TTFields (13). Importantly, as demonstrated in this study, this observation of added benefit with TTFields is also valid for the understudied, high-risk elderly patients who are ≥70 years of age, an age group in which the incidence of GBM is expected to increase (17).

TTFields therapy was safe and well tolerated in elderly patients with ndGBM. No difference in the incidence of systemic AEs or SAEs was observed between the 2 groups. About half of the patients in this analysis who were treated with TTFields plus TMZ combination experienced mild-to-moderate reversible and resoluble localized skin AEs beneath the arrays of the Optune^®^ device. These results are consistent with the overall phase 3 EF-14 trial population, as well as data from a large global post-marketing safety surveillance study of TTFields for high-grade gliomas that included 1,805 elderly patients with ndGBM (22). The safety of TTFields may be further improved by implementing practical strategies to prevent or treat dermatological AEs (23).

Radiotherapy and TMZ chemotherapy (vesicant) have been demonstrated to be cytotoxic and of noncontinuous (limited) administrative potential in this patient population. In comparison, TTFields therapy is a less invasive, well-tolerated, locoregional treatment modality that can be used continuously to target cancer cells (24). It has been most commonly associated with manageable, local scalp skin AEs related to array and hydrogel contact with skin. However, radiotherapy-related AEs may include local and systemic AEs, such as short- and long-term skin and scalp changes, hair loss, skin irritation, nausea and vomiting, seizures, hematological (low blood counts), fatigue, loss of hearing, and trouble with memory and speech (25). Similarly, TMZ chemotherapy has been associated with a variety of AEs, ranging from headaches and hair loss, to more toxic AEs such as myelosuppression (thrombocytopenia and leukopenia), infections, and gastrointestinal AEs (nausea and vomiting) (26). Based on these associated known toxic effects of radiotherapy and TMZ, adjuvant TTFields presents as a probable combinatorial partner to maximize elderly patient outcomes, without significant increases in systemic toxicity.

In addition, TTFields therapy did not impact HRQoL in elderly patients with ndGBM demonstrating tolerability. Importantly, the absence of difference in DFS and TTD between patients included in the maintenance TMZ and TTFields group and the control group previously observed in the complete study population in the EF-14 trial (14, 15) was maintained when analyzing the subset of patients who were ≥65 years of age. There were no differences observed between treatment groups for the 9 preselected items of the patient-reported EORTC QLQ-C30 and the QLQ-BN20 brain tumor module questionnaires. This finding indicates that the addition of TTFields to TMZ did not negatively impact HRQoL in elderly patients with ndGBM. These data were concordant with the overall EF-14 study population that also demonstrated no significant changes in HRQoL, except for more itchy skin, and hence supports the tolerability of TTFields in this population. Maintaining acceptable HRQoL is of particular importance in guiding treatment decisions for elderly patients with GBM (4), as QoL is generally prioritized in elderly patients with cancer compared to their younger counterparts (27–29).

These findings support a role for TTFields in the treatment of elderly patients with GBM, a large and therapeutically challenging subset of patients with GBM for whom there is no single clear SOC, provided that they fulfill the inclusion criteria of the EF-14 trial (4, 5, 30). In the phase 3 joint EORTC and National Cancer Institute of Canada Clinical Trials Group (NCIC) trial (3), (the Stupp trial, which established the current overall SOC for GBM), the survival benefit of adding concomitant and adjuvant TMZ to radiation therapy was seen to diminish with increasing age, with an HR of 0.78 (*P*=0.340) in patients who were 65–70 years of age. These data indicated that aggressive treatments should be carefully considered before use in elderly patients (17). Subsequent studies confirmed the benefit of radiation therapy in elderly patients with GBM and that hypofractionated radiation therapy (40 Gy in 15 fractions) is at least as effective as the standard schedule (60 Gy in 30 fractions) with improved tolerability (31, 32). The addition of concomitant and adjuvant TMZ to hypofractionated radiation therapy is supported by the phase 3 CCTG CE.6 trial, which reported a median OS of 9.3 months with radiation therapy plus TMZ *versus* 7.6 months with radiation alone (8). Studies of various schedules of radiation therapy *versus* TMZ alone have demonstrated comparable survival rates ranging from 6 to 10 months (32, 33). The significant improvement in median OS observed in this study compared to other trials can potentially be explained by patients being included only after completion of radiation therapy and confirmed absence of progression at that time point.

Of note, although a relatively good performance status was observed in this study, KPS scores as well as other baseline characteristics were balanced between both treatment groups. In addition, the reported dose-response relationship data further emphasize the validity of these positive survival outcomes in elderly patients with ndGBM. The study population had an overall better prognosis than patients of clinical trials that enrolled patients at the time of radiation therapy plus TMZ treatment initiation. Therefore, the findings in this study are focused on elderly patients who met inclusion criteria; hence, the best clinical assessment should be taken into account when extrapolating results to the entirety of the elderly patient population diagnosed with GBM. It is also worth noting that the drop-off in percentage of patients performing the HRQoL assessments over the 12-month follow-up period is a potential confounder. However, since the drop-off is similar in both treatment arms, this is likely due to the natural course of the disease. Regardless, the addition of TTFields to TMZ demonstrated clinical efficacy in elderly patients with ndGBM without negatively affecting HRQoL outcomes or introducing new safety concerns to the eligible study population.

Limitations of the EF-14 clinical trial have been previously addressed (13). In the present analysis, overall limitations include the non-prespecified, *post hoc* nature and the small sample sizes of both treatment groups due to the limited number of patients enrolled in the EF-14 trial who were ≥65 years of age. There is also a lack of available molecular data in ~20% of patients; 4% of TTFields plus TMZ-treated patients had an *IDH* mutation compared with 0% of the TMZ-treated patients; and only 1 patient had a 1p19q codeletion (TTFields plus TMZ group). Despite these limitations, the statistically significant improvement of the survival outcomes by the addition of TTFields to maintenance TMZ observed in this randomized study must, however, be stressed. These data strongly suggest that the addition of TTFields therapy in combination must be considered in all elderly patients with ndGBM who meet study eligibility criteria. TTFields therapy improved outcomes with limited safety concerns and without negatively impacting HRQoL outcomes, satisfying an unmet medical need in this patient population.

## Conclusions

In conclusion, in this subgroup analysis of elderly patients with ndGBM from the phase 3 EF-14 clinical trial, TTFields (200 kHz) plus TMZ combination improved OS and PFS survival outcomes compared to TMZ chemotherapy alone in patients who were ≥65 years of age. TTFields therapy was well tolerated with no negative impact on HRQoL or increase in systemic AEs. Also, TTFields daily usage of ≥75%, based on the duration of usage recommendations, was associated with additional and optimized survival benefits in this typically high-risk elderly patient population. These findings indicate that adjunctive therapy with TTFields is a safe and effective therapy in understudied, undertreated elderly patients with ndGBM, which is a great healthcare disparity, with no new safety signals. Overall, in elderly patients with ndGBM who are disease and treatment burdened, TTFields therapy demonstrated feasibility and safety as a viable combination partner that can be incorporated continuously to improve efficacy outcomes. Evidence supports the use of TTFields in this population without added risks and toxicity and with no negative impact on HRQoL. Hence, it can be concluded that compared to treatments that have been demonstrated to be toxic and with limited administration in this patient population, such as radiotherapy and TMZ, TTFields therapy is tolerable and can be used continuously in this entire patient population.

## Data Availability Statement

The raw data supporting the conclusions of this article will be made available by the authors, without undue reservation.

## Ethics Statement

The studies involving human participants were reviewed and approved by the institutional review boards or ethics committees of all participating centers in the original EF-14 trial (NCT00916409). The patients/participants provided their written informed consent to participate in this study.

## Author Contributions

All authors listed have made a substantial, direct, and intellectual contribution to the work and approved it for publication.

## Funding

The study was funded by Novocure.

## Conflict of Interest

ZR received research grants from and is a paid consultant of Novocure. AH received a research grant from Novocure, paid to the institution. AI received research grants and travel funding from Carthera, Leo Pharma and Novocure, research grants from Air Liquide, Nutritheragene, Sanofi, and Transgene, and travel funding from Leo Pharma, and served on received honorarium for advisory boards from Novocure and Leo Pharma. J-JZ received funding paid to the institution from Boston Biomedical Sumitomo Dainippon Pharma Global Oncology, Novocure, Inc., and NRG consortium of National Cancer Institute (NCI) and an honorarium from the Hong Kong Precision Oncology Society.

The remaining authors declare that the research was conducted in the absence of any commercial or financial relationships that could be construed as a potential conflict of interest.

The authors declare that this study received funding from Novocure. The funder had the following involvement with the study: design and conduct of the study; collection, management, analysis, andinterpretation of the data; and preparation and review of this manuscript.

## Publisher’s Note

All claims expressed in this article are solely those of the authors and do not necessarily represent those of their affiliated organizations, or those of the publisher, the editors and the reviewers. Any product that may be evaluated in this article, or claim that may be made by its manufacturer, is not guaranteed or endorsed by the publisher.
