# Corrigendum: Efficacy and Safety of Tumor Treating Fields (TTFields) in Elderly Patients With Newly Diagnosed Glioblastoma: Subgroup Analysis of the Phase 3 EF-14 Clinical Trial

**DOI:** 10.3389/fonc.2022.902929

**Published:** 2022-04-12

**Authors:** Zvi Ram, Chae-Yong Kim, Andreas F. Hottinger, Ahmed Idbaih, Garth Nicholas, Jay-Jiguang Zhu

**Affiliations:** ^1^Department of Neurosurgery, Tel Aviv Medical Center and Tel Aviv University School of Medicine, Tel Aviv, Israel; ^2^Department of Neurosurgery, Seoul National University College of Medicine, Seoul, South Korea; ^3^Department of Clinical Neuroscience, CHUV Lausanne University Hospital & University of Lausanne, Lausanne, Switzerland; ^4^Service de Neurologie 2-Mazarin, Sorbonne Université, Inserm, CNRS, UMR S 1127, Institut du Cerveau, ICM, AP-HP, Hôpitaux Universitaires La Pitié Salpêtrière—Charles Foix, Service de Neurologie 2-Mazarin, Paris, France; ^5^Department of Medicine, University of Ottawa, Ottawa, ON, Canada; ^6^Department of Neurosurgery, University of Texas Health Science Center at Houston, Houston, TX, United States

**Keywords:** elderly patients, newly diagnosed glioblastoma, TTFields, Tumor Treating Fields, phase 3 clinical trial, efficacy and safety, quality-of-life, temozolomide

In the original article, there was a mistake in **Figure 1** as published. An entry was labelled as “*No adherence”* rather than “*Disease progression”*. The corrected **Figure 1** appears below.

**Figure 1:**
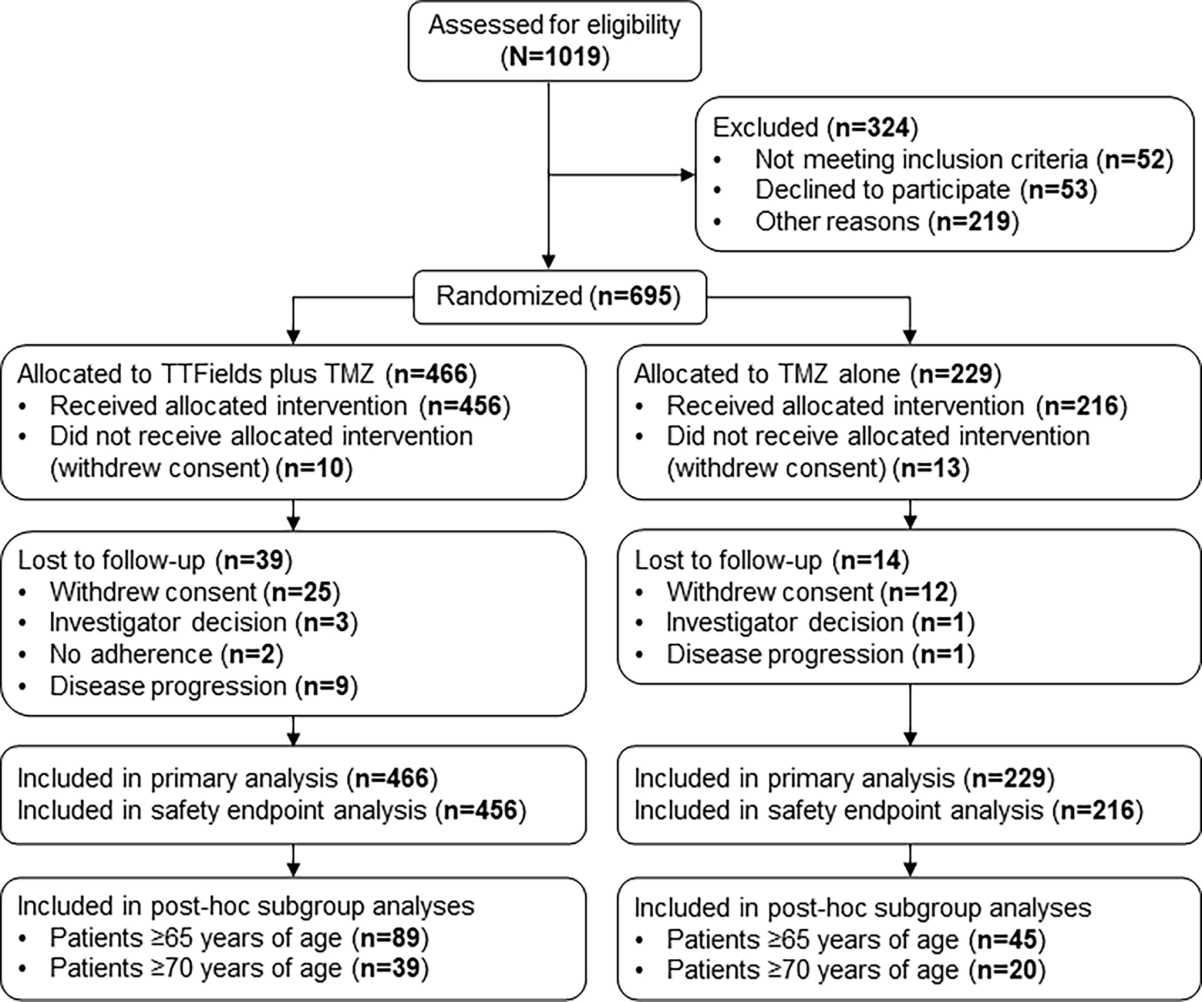
CONSORT Diagram. Updated from Stupp R, Taillibert S, Kanner A, Read W, Steinberg DM, Lhermitte B, et al. Effect of tumor-treating fields plus maintenance temozolomide vs maintenance temozolomide alone on survival in patients with glioblastoma a randomized clinical trial. JAMA. (2017) 318:2306–16 TMZ, temozolomide; TTFields, Tumor Treating Fields.

In the original article, there was also a mistake in **Table 2** as published. Incorrect data appears for *Duration of TTFields therapy, months, median (range)* and *TTFields daily usage ≥75%, n (%)* for *TTFields plus TMZ*. The corrected **Table 2** appears below.

**Table 2:** Baseline characteristics in TTFields (200 kHz) plus TMZ combination versus TMZ monotherapy groups for patients ≥70 years of age.

Characteristics	TTFields plus TMZ (n=39)	TMZ alone (n=20)	*P* value*
Age, years, median (range)	74 (70–83)	73 (70–80)	0.186
Sex, n (%)			0.957
Male	29 (74)	15 (75)	
Female	10 (26)	5 (25)	
Corticosteroid therapy, n (%)	12 (31)	5 (25)	0.643
Extent of resection, n (%)			0.312
Biopsy	7 (18)	1 (5)	
Partial resection	13 (33)	6 (30)	
Gross total resection	19 (49)	13 (65)	
*MGMT* tissue available and tested, n (%)			0.443
Methylated	16 (46)	4 (27)	
Unmethylated	15 (43)	9 (60)	
Invalid	4 (11)	2 (13)	
*IDH1^R132H^* tissue available and tested, n (%)	24 (62)	10 (50)	0.512
Positive	1 (4)	0 (0)	
Negative	23 (96)	10 (100)	
EGFR tissue available and tested, n (%)	26 (67)	10 (50)	0.529
Amplified	10 (38)	5 (50)	
Not amplified	16 (62)	5 (50)	
Chromosomes 1p and 19q tissue available and tested, n (%)	24 (62)	10 (50)	0.241
Codeletion	0 (0)	0 (0)	
Loss 1p only	0 (0)	0 (0)	
Loss 19q only	0 (0)	1 (10)	
Retained	23 (96)	9 (90)	
Invalid	1 (4)	0 (0)	
KPS,^a^ median (range)	85 (60–100)	90 (70–100)	
Time from diagnosis to randomization, months, median (range)	3.7 (2.6–5.1)	3.9 (2.8–5.4)	0.268
Time from last day of radiotherapy to randomization, days, median (range)	35 (23–49)	42 (29–50)	0.016
TMZ cycles until first tumor progression, n, median (range)	6 (1–15)	6 (1–12)	0.441
Time from randomization to TTFields initiation, days, median (range)	5 (1–13)	*NA*	
Duration of TTFields therapy, months, median (range)	6.9 (0–40)	*NA*	*NA*
TTFields daily usage ≥75%, n (%)	19 (41)	*NA*	*NA*

EGFR, epidermal growth factor receptor gene; IDH1^R132H^, isocitrate dehydrogenase gene 1 R132H mutation site; KPS, Karnofsky Performance Score; MGMT, O^6^-methylguanine-DNA-methyltransferase gene; n, number of patients; NA, not applicable; TMZ, temozolomide; TTFields, Tumor Treating Fields.

a Karnofsky Performance Score is measured from 0 to 100 in 10-point bins. A higher score represents better performance status.

*Chi-squared test for percentage values and T test for means values.

Total percentage sums may not equal 100 or total percent of a patient subpopulation due to rounding to nearest integer.

In the original article, there was a further mistake in **Figure 3C** as published. There was an error in *KPS, median (range)* for *TTFields (200 kHz) ≥75% Daily Usage*. The corrected **Figure 3** appears below.

**Figure 3:**
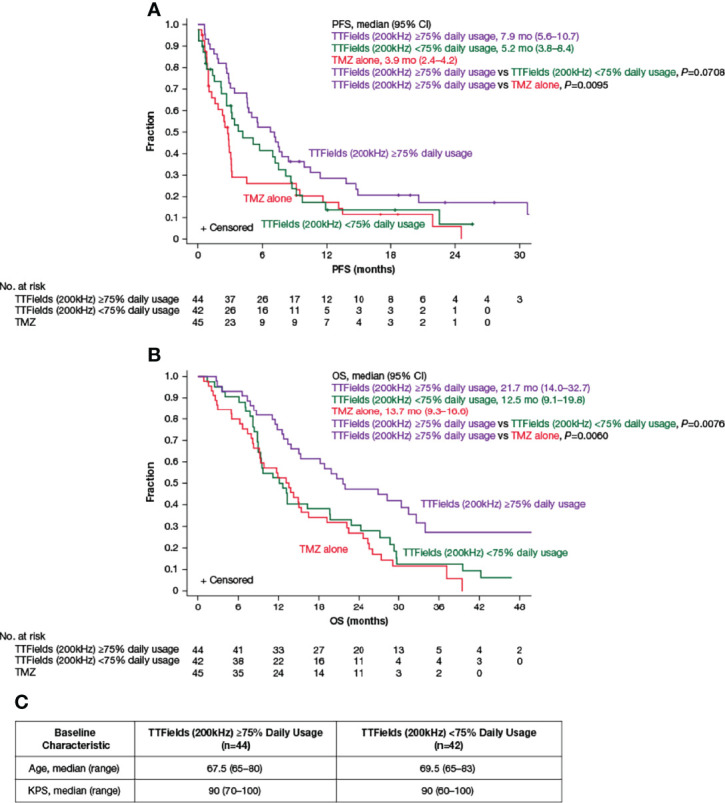
Kaplan–Meier curves of **(A)** median PFS and **(B)** median OS of TTFields plus TMZ combination with TTFields daily usage of ≥75% (purple lines) compared to TTFields daily usage of <75% (green lines) and TMZ alone (red lines). **(C)** Baseline age and KPS of patients ≥65 years of age with TTFields daily usage of ≥75% and <75%. CI, confidence interval; KPS, Karnofsky Performance Status; mo, month; no, number; OS, overall survival; PFS, progression-free survival; TMZ, temozolomide; TTFields, Tumor Treating Fields.

The authors apologize for these errors and state that this does not change the scientific conclusions of the article in any way. The original article has been updated.

## Publisher’s Note

All claims expressed in this article are solely those of the authors and do not necessarily represent those of their affiliated organizations, or those of the publisher, the editors and the reviewers. Any product that may be evaluated in this article, or claim that may be made by its manufacturer, is not guaranteed or endorsed by the publisher.

