# Erratum: Corrigendum: Efficacy and Safety of Tumor Treating Fields (TTFields) in Elderly Patients With Newly Diagnosed Glioblastoma: Subgroup Analysis of the Phase 3 EF-14 Clinical Trial

**DOI:** 10.3389/fonc.2022.952053

**Published:** 2022-06-07

**Authors:** Frontiers Production Office

**Affiliations:** Frontiers Media SA, Lausanne, Switzerland

**Keywords:** elderly patients, newly diagnosed glioblastoma, TTFields, Tumor Treating Fields, phase 3 clinical trial, efficacy and safety, quality-of-life, temozolomide

Due to a production error, there was an error in the title. The corrected title appears above. The publisher apologizes for this mistake. The original version of this article has been updated.

